# Exploring the Role and Mechanism of pAMPK*α*-Mediated Dysregulation of Brf1 and RNA Pol III Genes

**DOI:** 10.1155/2021/5554932

**Published:** 2021-04-20

**Authors:** Teng Wu, Dongkun Zhang, Mingen Lin, Lihong Yu, Ting Dai, Shuai Li, Fenghai Yu, Lei Lu, Liling Zheng, Shuping Zhong

**Affiliations:** ^1^GMU-GIBH Joint School of Life Sciences, Guangzhou Medical University, Guangzhou, China; ^2^Department of Thoracic Surgery, Guangdong Provincial People's Hospital, Guangdong Academy of Medical Sciences, Guangzhou, China; ^3^The First Affiliated Hospital of Shantou University Medical College, China; ^4^Keck School of Medicine, University of Southern California, Los Angeles, CA, USA; ^5^First Hospital of Quanzhou Affiliated to Fujian Medical University, China

## Abstract

TF IIB-related factor 1 (Brf1) is a key transcription factor of RNA polymerase III (Pol III) genes. Our early studies have demonstrated that Brf1 and Pol III genes are epigenetically modulated by histone H3 phosphorylation. Here, we have further investigated the relationship of the abnormal expression of Brf1 with a high level of phosphorylated AMPK*α* (pAMPK*α*) and explored the role and molecular mechanism of pAMPK*α*-mediated dysregulation of Brf1 and Pol III genes in lung cancer. Brf1 is significantly overexpressed in lung cancer cases. The cases with high Brf1 expression display short overall survival times. Elevation of Brf1 expression is accompanied by a high level of pAMPK*α*. Brf1 and pAMPK*α* colocalize in nuclei. Further analysis indicates that the carcinogen MNNG induces pAMPK*α* to upregulate Brf1 expression, resulting in the enhancement of Pol III transcription. In contrast, inhibiting pAMPK*α* decreases cellular levels of Brf1, resulting in the reduction of Pol III gene transcription to attenuate the rates of cell proliferation and colony formation of lung cancer cells. These outcomes demonstrate that high Brf1 expression reveals a worse prognosis in lung cancer patients. pAMPK*α*-mediated dysregulation of Brf1 and Pol III genes plays important roles in cell proliferation, colony formation, and tumor development of lung cancer. Brf1 may be a biomarker for establishing the prognosis of lung cancer. It is a new mechanism that pAMPK*α* mediates dysregulation of Brf1 and Pol III genes to promote lung cancer development.

## 1. Introduction

Lung cancer is a common malignant tumor. In recent years, the incidence of lung cancer in China has increased [1, 2]. It is a malignant tumor with the highest morbidity and mortality in the country. Based on cytological and histological characterization, lung cancer is divided into small-cell lung cancer (SCLC) and non-small-cell lung cancer (NSCLC). SCLC and NSCLC account for 15% and 80-85% of cases, respectively [3, 4]. SCLC is a heterogeneous neoplastic disease characterized by aggressiveness, rapid growth of cancer cells, and easy metastases [5], while NSCLC is a kind of epithelial malignant disease apart from SCLC. NSCLC is not sensitive to chemotherapy, which is mainly performed by surgical resection with curative intent. The causes of lung cancer are complex, such as environmental pollution and genetic and epigenetic changes. The exact mechanism of lung cancer is not fully understood. Lung carcinogenesis involves multiple mechanisms: oncogene activation, such as K-Ras [6]; inactivation of tumor suppressor genes (LKB1) [7]; epidermal growth factor receptor (EGFR) mutation and amplification [8]; inhibition of immune system activity [9, 10]; and epigenetic alterations (DNA methylation, histone tail modifications, and small RNAs) [11]. To date, no studies elucidate the roles of dysregulation of TF IIB-related factor 1 (Brf1) and its target genes, RNA polymerase III-dependent genes (Pol III genes) in lung cancer development, whereas dysregulation of Brf1 and Pol III genes is tightly related to tumor development.

Brf1 is a key transcription factor of tRNAs and 5S rRNA, which are Pol III genes. Brf1 specifically modulates the transcription of these genes [12–15]. Dysregulation of tRNAs and 5S rRNA genes is directly linked to cell transformation and tumorigenesis [16–18], and it also helps to enhance the cellular ability of protein synthesis for cell growth, proliferation and transformation, and tumor development. Increasing Brf1 expression elevates the activities of tRNA and 5S rRNA genes. In contrast, repressing Brf1 decreases the activity of these genes and inhibits cell proliferation and tumor development [17, 18]. Recent studies of ours and others indicate that Brf1 overexpression is founded in hepatocyte carcinoma (HCC), breast cancer, gastric carcinoma, and prostate cancer [19–22]. This shows that Brf1 plays an important role in human cancer development and tumor growth. However, it remains to be detected if Brf1 expression is increased in human cases of lung cancer and what is the significance of its expressional status in the diagnosis and prognosis of this disease.

5′ AMP-activated protein kinase or 5′ adenosine monophosphate-activated protein kinase (AMPK) is an enzyme. AMPK is composed of three subunits (*α*, *β*, and *γ*) to form a heterotrimeric protein complex, and these subunits play critical roles in its activity and stability [23]. AMPK increases glucose uptake and inhibits the synthesis of fatty acids, cholesterol, and triglycerides and promotes fatty acid uptake and *β*-oxidation [24]. It indicates that AMPK plays critical roles in the regulation of energy metabolism. AMPK is activated in low-energy cellular states by phosphorylating its subunits [25]. AMPK is a primary component of the LKB1 downstream pathway, while mutations of LKB1 are found in over 20% of patients with NSCLC and frequently associated with activating K-RAS mutations [26–28]. Tumor suppressor LKB1 mediates AMPK activity. As enhancements of Brf1 and Pol III gene activities are tightly related to human cancers, this suggests that AMPK activation may involve the modulation of Brf1 and Pol III gene transcription to increase cell proliferation and promote tumor development.

Our earlier studies have demonstrated that alcohol increases Brf1 expression in tissue culture and animal models, which facilitates cell proliferation and transformation, and tumor formation [15, 17, 19, 20, 29–32]. Chronic alcohol consumption results in the production of acetaldehyde and CYP2E1 induction (Cytochrome P450 2E1) [14]. Acetaldehyde is a by-product of alcohol metabolism catalyzed by ADH (alcohol dehydrogenase), which has direct mutagenic and carcinogenic effects *in vitro* and *in vivo* [14, 33]. CYP2E1 is associated with the release of ROS (reactive oxygen species) and conversion of procarcinogens to carcinogens [14]. Alcohol exposure increases cellular production of ROS, causing cellular stress to result in tissue injury and diseases [14]. ROS-induced oxidative stress activates the JNK1 pathway to increase Brf1 expression [31]. A recent study also indicates that levels of ROS of lung cancer cells are associated with the alteration of pAMPK*α* [34], while AMPK activation is associated with protein synthesis [24, 25], which is controlled by Brf1 and Pol III genes. This implies that ROS and AMPK are potentially involved in Brf1 expression, which may be associated with lung cancer.

Here, we report, for the first time, that Brf1 expression is enhanced in human cases of lung cancer. High expression of Brf1 reveals short survival times (*p* = 0.0013). Activation of AMPK increases Brf1 promoter activity to upregulate Brf1 expression, resulting in elevation of tRNAs and 5S rRNA transcription. Repression of AMPK decreases cellular levels of Brf1, tRNAs, and 5S rRNA expression, leading to reducing the rates of cell proliferation and colony formation. Brf1 and pAMPK*α* are colocalized in lung cancer cell nuclei, which maybe synergistically regulate the transcription of Pol III genes. The study identifies a new pathway, pAMPK*α*, which modulates Brf1, tRNAs, and 5S rRNA transcription in lung cancer cells. This shows that both Brf1 and pAMPK*α* play an important role in lung cancer.

## 2. Materials and Methods

### 2.1. Human Tissue Samples

The 226 samples of paraffin-embedded, archived lung cancer tissue samples used in this study were histopathologically and clinically diagnosed at the Guangdong Provincial People's Hospital and Guangdong Academy of Medical Sciences and Shanghai Outdo Biotech Ltd after obtaining written informed consent and in accordance with the Institutional Review Board and the Declaration of Helsinki. No patient received any chemo- or radiotherapy prior to surgery [35]. The patients were followed up regularly after the operation at three-month intervals [35]. Eight freshly collected lung cancer tissues and matched adjacent nontumoral lung tissues were frozen and stored in liquid nitrogen until required for protein extraction [35]. Informed consent was obtained from each patient, and the study was approved by the Institute Research Ethics Committee of Guangdong General Hospital, Guangdong Academy of Medical Sciences (ID number: No. GDREC2016175H(R2)).

### 2.2. Cell Lines and Reagents

The lung cancer cell lines A549 and H1975, and normal human bronchial epithelial cell line 16HBE were purchased from the American Type Culture Collection (Manassas, VA) and cultured in RPMI 1640 containing 10% FBS, 100 IU/mL penicillin, and 100 *μ*g/mL streptomycin (HyClone, Utah, USA). All cells were tested for negative mycoplasma contamination and authenticated based on short tandem repeat fingerprinting before use. AMPK inhibitor, S7306 (Dorsomorphin (Compound C) 2HCl, Cat No. S7306), was purchased from Selleck and dissolved in sterile water. N-Methyl-N′-nitro-N-nitrosoguanidine (MNNG) was purchased from Accu. Standard, Inc (Cat No. R-081N) and dissolved in DMSO. Brf1 antibody was from Bethyl Laboratories Inc (Cat No. A301-228A). The MTT assay kit was from Boster Biotech (Cat No. AR1156).

### 2.3. Immunohistochemistry

We performed immunohistochemical staining with Brf1 antibody (1 : 200). Its details were described in our previous study [20, 30].

The levels of Brf1 immunostaining were evaluated independently by two pathologists who were blinded to the survival outcomes of the participants based on the proportion of positively stained tumor cells (stain area) and the intensity of staining [20]. The immunostaining results were performed by multiplying the staining intensity by the stained area (staining index (SI)) as previously described [30, 36, 37]. The Brf1 expression levels in lung cancer lesions were determined by the SI, which was 0, 1, 2, 3, 4, 6, 9, or 12. An optimal cutoff value was identified as follows: an SI score of ≥6 was used to define tumors as high Brf1 expression, and an SI score of ≤4 as low [30, 36, 37].

### 2.4. Immunoblot Analysis

Tissue samples of lung cancer were ground into powder with liquid nitrogen and lysed in lysis buffer with phosphatase and protease inhibitors [20]. Lung cancer cells were treated with 4 *μ*M MNNG or 10 *μ*M AMPK inhibitor, S7306, to extract total cell lysates, and the protein concentrations were measured using the BCA Protein Assay (Thermo Fisher Scientific, Cat No. 23225) [35]. Equal amounts of cell protein were subjected to electrophoresis in SDS-PAGE gels and then transferred to PVDF membranes (Millipore) for antibody blotting [35]. Bound primary antibody was visualized using horseradish peroxidase-conjugated secondary antibodies (Proteintech, Cat No. SA00001-1 or SA00001-2) and enhanced chemiluminescence reagents (Beyotime, Cat No. P0018S). Antibodies used in our study were as follows: Brf1 (1 : 2000), pAMPK*α* (CST, Cat No. 2535S, 1 : 1000), AMPK*α* (CST, Cat No. 2793S, 1 : 1000), and *β*-actin (Proteintech, Cat No. 20536-1-AP, 1 : 5000). All of the experiments were repeated at least three times [20].

### 2.5. Quantitative Real-Time PCR

Total RNAs were isolated with TRIzol (Invitrogen, Cat No.15596026) following the manufacturer's protocol. Then, reverse transcription was performed (Takara, Cat No. RR036A). Target mRNA levels were determined by performing RT-qPCR with a TB Green® Premix Ex Taq™ II (Tli RNaseH Plus) kit (Takara, Cat No. RR820A). GAPDH expression was used for normalization. The sequences of the primers were described previously [17, 18].

### 2.6. Immunofluorescence

For colocation detection, the lung cancer cells were fixed for 30 min in 4% formaldehyde/PBS, washed with 0.2% Triton-X 100/PBS [37]. The cell slices were blocked with 1% BSA/PBS for 1 h at room temperature and were incubated with Brf1 antibodies (1 : 200) overnight at 4°C and then incubated with anti-rabbit IgG (Proteintech, Cat No. SA00013-4, 1 : 200) for 1 h as secondary antibodies. The cell slices were immersed in 1x PBS and heated in a microwave oven at 42°C for 3 min to remove nonspecific bindings. Subsequently, pAMPK*α* antibodies (1 : 200) were incubated overnight at 4°C and then incubated with anti-rabbit IgG (Proteintech, Cat No. SA00013-2, 1 : 200) for 1 h as secondary antibodies [20]. Cell nuclei were counterstained with 2 *μ*g/mL DAPI (Biofroxx, Cat No. 1155MG010) for 5 min, and the slides were mounted in an antifade reagent (Life Technologies, Cat No. P36934). The cells were visualized under a fluorescence microscope (ZEISS, Germany) [30, 37].

### 2.7. siRNA Transfection and Brf1-Luc Reporter Assays

For siRNA knockdown, Brf1 siRNAs, AMPK*α* siRNA, and a control siRNA (mismatch RNA: mmRNA) were purchased from RiboBio. The sequences of primers and siRNAs used were as previously described [17, 18]. Transfections were performed using Lipofectamine 3000 and OPTI-MEM reagent (Life Technologies, Cat No. L3000015 and 11058021) when cells were approximately 40% confluent and transfected according to the manufacturer's instruction [37]. For Brf1-Luc promoter activity, cells were transfected with 0.5 *μ*g of the Brf1-Luc report constructs for 48 h. Cells were starved in FBS-free RPMI 1640 for 4 h and treated with different concentrations of MNNG for another 2 h. Cell pellets were dissolved in Promega reporter lysis buffer. The luciferase activities of these lysates were determined by a luminometer and the Promega Luciferase Kit (Promega, Cat No. E1910). The luciferase activities of the lysates were normalized to their protein amounts as described [31, 32]. The changes in luciferase activity were compared to the luciferase activity in the absence of MNNG. Means ± SE is at least three independent experiments.

### 2.8. Colony Formation Assay

A549 cells were transfected with mismatch RNA (mmRNA), Brf1 siRNAs, and AMPK*α* siRNAs as described [31]. The transfected A549 cells (1 × 10^4^ cells/well in 6-well plates) were mixed with equal volumes of 0.7% soft agar dissolved in RPMI 1640 (10% FBS) with or without 4 *μ*M MNNG and layered in triplicate onto 0.7% (RPMI 1640, 10% FBS) solidified agar. Cells were fed fresh complete media with MNNG twice weekly. Colonies were counted 2–3 weeks or longer after under a microscope and photographed as described previously [38].

### 2.9. Statistical Analyses

We carried out statistical analysis with Student's *t*-test, ANOVA and Tukey's multiple comparisons test, Kaplan-Meier and log-rank test, ROC curve, and Cox analysis. The details of the statistical analysis were described previously [37].

## 3. Results

### 3.1. Brf1 Expression in Lung Cancer Tissues and Its Significance

**Figure 1:**
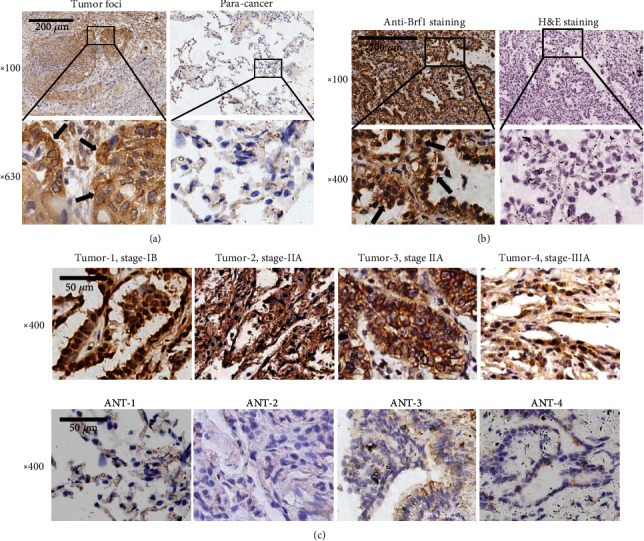
Immunohistochemical staining of Brf1 in lung cancer. (a) Comparison of Brf1 staining in tumor foci tissue with paracancer tissue (para-can) of lung cancer patients. Strong staining signals of Brf1 expression are seen in tumor foci of lung cancer (a, left panel). Weak signals of Brf1 staining are detected in para-can tissue of this disease (a, right panel). Top panel: 100x magnification (scale bar = 200 *μ*m); bottom panel: 630x magnification. (b) Comparison of Brf1 IHC and H&E staining in the same cases of lung cancer. IHC staining about the signals of Brf1 expression in both cytoplasm and nuclei of tumor tissues of lung cancer; (b, left panel) H&E staining of tumor tissues of lung cancer; (b, right panel) 100x magnification (scale bar = 200 *μ*m); 400x magnification. (c) Comparison of Brf1 expression in tumor foci with adjacent noncancerous tissue (ANT). The levels of Brf1 expression were detected in four lung cancer lesions (c, upper panel) and their paired ANT (c, lower panel). 400x magnification (scale bar = 50 *μ*m). The results indicate that Brf1 expression was increased in the tumor tissues at different stages of lung cancer, compared to noncancerous tissues, ANT.

**Figure 2:**
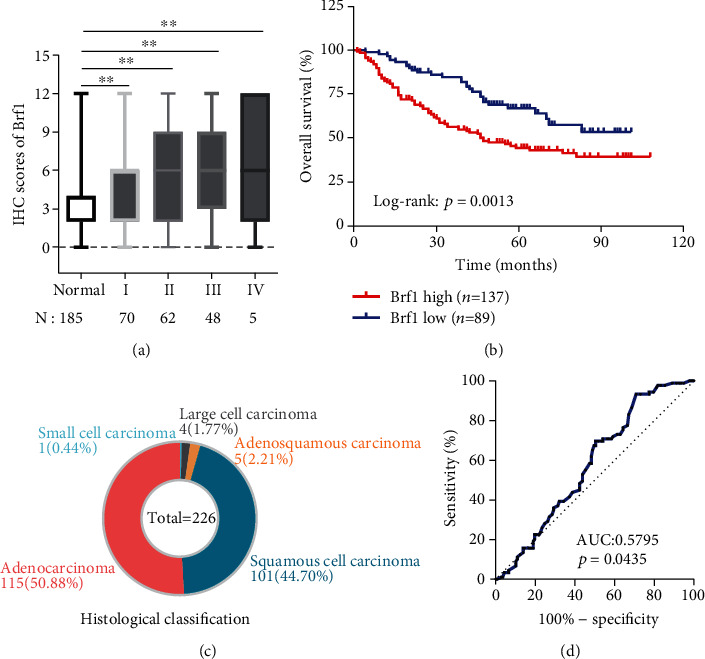
High Brf1 expression correlated with a poor prognosis of lung cancer. (a) The IHC staining scores of Brf1 expression. 185 cases in paired different clinical stages of lung cancer tumor tissues show high Brf1 expression, compared to low expression of Brf1 expression corresponding to normal tissues of these cases (*N* = 185). (b) 226 cases of human lung cancer patients were performed for Kaplan-Meier analysis of the overall survival period. Lung cancer patients (*N* = 226) with low versus high expression of Brf1 (Kaplan-Meier analysis with the log-rank test, *p* < 0.01). (c) Histological classification of the 226 cases of lung cancer. (d) ROC curve analysis. The result reveals that patients with high Brf1 expression display short survival times.

Brf1 plays an increasingly important role in human cancers. Emerging evidence indicates that Brf1 expression is elevated in the cases of human liver, breast, gastric, and prostate cancers [19–22]. To test Brf1 expression in the cases of lung cancer, we utilized the samples of this disease to determine the levels of Brf1 expression in tumor foci and paracarcinoma tissues by immunohistochemistry (IHC) staining. The result indicates that a strong signal of Brf1 was detected in tumor foci tissue (Figure 1(a), left panel), while a very weak reaction of Brf1 with its antibody was observed in paracarcinoma tissue (Figure 1(a), right panel). The overall reaction of Brf1 staining in tumor foci of lung cancer is markedly higher than that in paracarcinoma tissue (Figure 1(a)).  Figure 1(b) reveals the results of Brf1 staining (Figure 1(b), left panel) and H&E staining (Figure 1(b), right panel). There are strong signals of Brf1 in the cytoplasm and nuclei of the tumor tissues of lung cancer (Figure 1(c), upper panel), whereas there are very weak or no signal of Brf1 expression in the corresponding adjacent noncancerous tissues (ANT) (Figure 1(c), lower panel). Results indicate that Brf1 expression in both early and advanced stages of lung cancer reveals strong signals in tumor tissues (Figure 1(c), upper panel). To further detect the relationship between Brf1 overexpression and clinical grades of lung cancer, we analyzed Brf1 expression in different stages of this disease. The paired analysis of Brf1 expression in tumor and normal tissues of lung cancer indicates that the levels of Brf1 expression in different clinical stage tumor tissues are significantly higher than that of normal tissues (Figure 2(a)). This implies that once tumorigenesis happens in the lung tissue, Brf1 expression will be significantly increased.

Furthermore, we determined the relationship between Brf1 expression and the overall survival period of lung cancer patients. The clinical information for the patients does not reveal any significant correlation of Brf1 expression with age, sex, and classification (Figure 2(c), Tables 1 and 2). We used four levels of intensity of Brf1 expression: negative staining, weak staining, moderate staining, and strong staining in these cases of lung cancer (Figures S1A and S1B). The strong staining of Brf1 in lesion tissues with staining index (SI) ≥ 6 was classified as high expression of Brf1. The result shows that about 60% of cases of lung cancer with high Brf1 expression display significant short overall survival times (Figure 2(b)). In addition, we also performed the ROC (receiver operator characteristic) curve analysis. The AUC result indicates that the accuracy of high Brf1 expression is a little low as a diagnostic biomarker for lung cancer (Figure 2(d)). Together, these studies indicate that Brf1 is overexpressed in lung cancer patients, and as a result, high expression of Brf1 reveals a worse prognosis. Brf1 may be a biomarker for the prognosis of the disease.

### 3.2. The Relationship between AMPK Activation and Brf1 Expression in Lung Cancer

**Figure 3:**
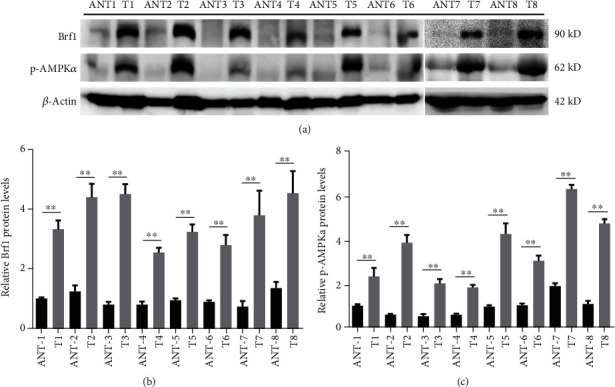
The relationship of AMPK*α* activation with Brf1 high expression in lung cancers. (a) Immunoblotting analysis of Brf1 and pAMPK*α* in 8 paired lung cancer tissues (T1: adenocarcinoma IIIB stage; T2: squamous cell carcinoma IIB stage; T3: adenocarcinoma IIA stage; T4: adenocarcinoma IA3 stage; T5: squamous cell carcinoma IIA stage; T6: adenocarcinoma IA1 stage; T7: adenocarcinoma IA3 stage; T8: adenocarcinoma IIIA stage). (b, c) The quantification of cellular levels of Brf1 (b) and pAMPK*α* (c) in the indicated lung cancer tissues was calculated and compared with the corresponding ANT. *p* values were determined by a two-tailed *t*-test. Data are presented as the mean ± SD of at least three independent experiments. ^∗^*p* < 0.05, ^∗∗^*p* < 0.01.

**Figure 4:**
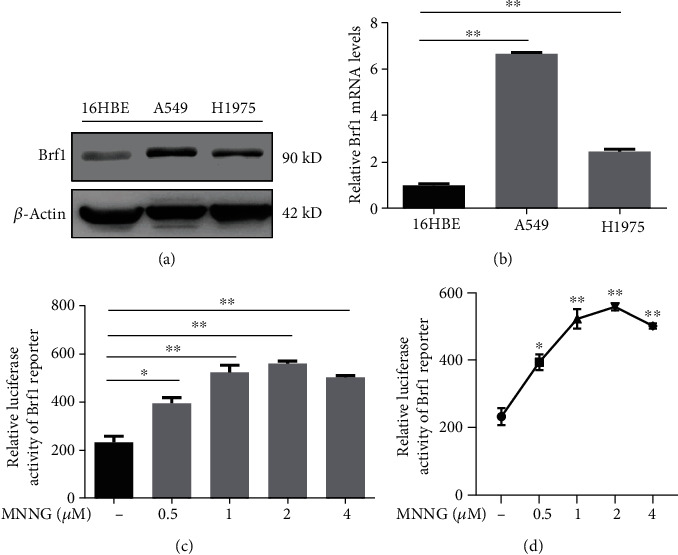
Brf1 expression in the cell lines of lung cancer and Brf1 promoter activity. (a) Immunoblotting analysis of Brf1 protein levels in the normal human bronchial epithelial cell line (16HBE) and lung cancer cell lines (A549 and H1975). (a) Is a representative result of immunoblotting. (b) RT-qPCR analysis of Brf1 mRNA levels in lung cancer cell lines (A549 and H1975) and nontumor line, 16HBE. (c, d) Brf1 promoter-luciferase activity. The A549 cells were transfected with 0.5 *μ*g Brf1-Luc plasmids. Luciferase assay indicates that the carcinogen MNNG increases the activity of the Brf1 promoter. All error bars represent the SD of at least three independent experiments. *p* values were determined by a two-tailed *t*-test. ^∗^*p* < 0.05, ^∗∗^*p* < 0.01.

The tumor suppressor gene, LKB1, is an upstream component and regulator of AMPK activation, but it is the most frequently mutated gene in lung cancer [26–28]. This suggests that mutant LKB1 loses its tumor suppressor function, leading to lung cancer development. Activated AMPK may be detected by its phosphorylation antibody in lung cancer samples, while the activated AMPK may mediate Brf1 expression and Pol III gene transcription. To test this hypothesis, we collected samples of human lung cancer to detect the levels of Brf1 proteins and phosphorylated AMPK*α* (pAMPK*α*) by immunoblot analysis and explore the correlation of the levels of pAMPK*α* with Brf1 expression. The results indicate that Brf1 expression is significantly increased in tumor tissues of lung cancer, compared to adjacent noncancerous tissue (ANT) samples in the same case (Figure 3(a)). Interestingly, pAMPK*α* levels in the tumor tissues are also much higher than those in corresponding ANT samples (Figure 3(a)). The quantitation of the immunoblot results of these samples indicates that the cellular levels of Brf1 (Figure 3(b)) and pAMPK*α* (Figure 3(c)) in tumor tissues are significantly higher than those in corresponding ANT samples. In addition, we also determined the cellular levels of Brf1 in the bronchial epithelial cells and lung cancer cell lines of humans. The immunoblot analysis reveals that the cellular levels of Brf1 in lung cancer cell lines, A549 and H1975, are higher than those in no lesion bronchial epithelial cell line, 16HBE (Figure 4(a)). We also determined the levels of Brf1 mRNA in the cell lines by RT-qPCR. The results indicate that the levels of Brf1 mRNA in A549 and H1975 cell lines are dramatically higher than those in bronchial epithelial cells (Figure 4(b)). High Brf1 expression is consistent with pAMPK*α* elevation in tumor tissues of lung cancer. We established the Brf1 promoter-luciferase reporter construct to test whether AMPK mediates Brf1 promoter activity. Figures 4(c) and 4(d) indicate that MNNG increases Brf1 transcription.

### 3.3. pAMPK*α* Mediates Brf1 Expression Resulting in the Enhancement of Pol III Gene Transcription

**Figure 5:**
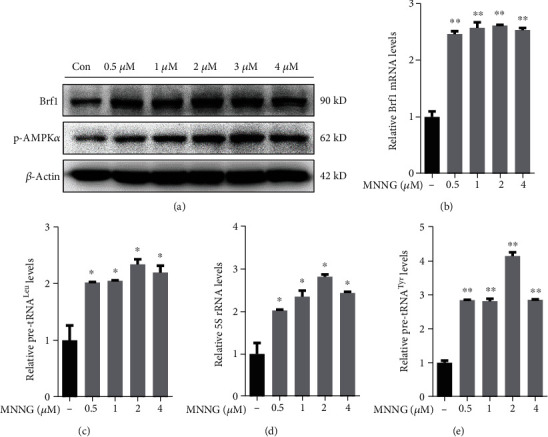
MNNG induces Brf1 expression and Pol III gene transcription. A549 cells were treated with different doses of the carcinogen MNNG. The resultant cell lysis and total RNA were extracted from the cells for immunoblotting analysis and RT-qPCR. (a) Immunoblotting analysis of cellular levels of Brf1 and pAMPK*α*. (b–e) RT-qPCR. Brf1 mRNA (b) and transcription levels of tRNA^Leu^ (c), 5S rRNA (d), and tRNA^Tyr^ (e). The results indicate that MNNG activated pAMPK*α* and enhanced Brf1 expression and Pol III gene transcription. All error bars represent the SD of at least three independent experiments. *p* values were determined by a two-tailed *t*-test. ^∗^*p* < 0.05, ^∗∗^*p* < 0.01.

Given Brf1 expression with high levels of pAMPK*α* in the cases of human lung cancer (Figure 3), we further determine whether pAMPK*α* mediates Brf1 expression and Pol III gene transcription. A549 cells were cultured in 10% FBS/RMPI 1640 medium to 80-85% confluence and starved in FBS-free medium for 4 h. The cells were treated with different doses of carcinogen, MNNG. The resultant lysates and RNA were used to detect the protein and mRNA levels of Brf1 and pAMPK*α*. The results indicate that MNNG markedly induced pAMPK*α* (Figure 5(a), middle). More interestingly, MNNG also increases the accompanied cellular levels of Brf1 proteins and mRNAs in various MNNG doses in A549 cells (Figures 5(a), top and 5(b)). Since MNNG enhances Brf1 promoter activity in A549 cells (Figures 4(c) and 4(d)), similar results are also observed in the lung cancer cell line, H1975 (Figures S2 and S3C). Thus, we further determined whether MNNG-activated AMPK, pAMPK*α*, affects the target genes of Brf1. The results show that MNNG increases Pol III gene, tRNA^Leu^, 5S rRNA, and tRNA^Tyr^ transcription in A549 (Figures 5(c)–5(e)) and H1975 cell lines (Figures S2B and S2C). This points out that pAMPK*α* really modulates the activities of Brf1 and Pol III genes.

**Figure 6:**
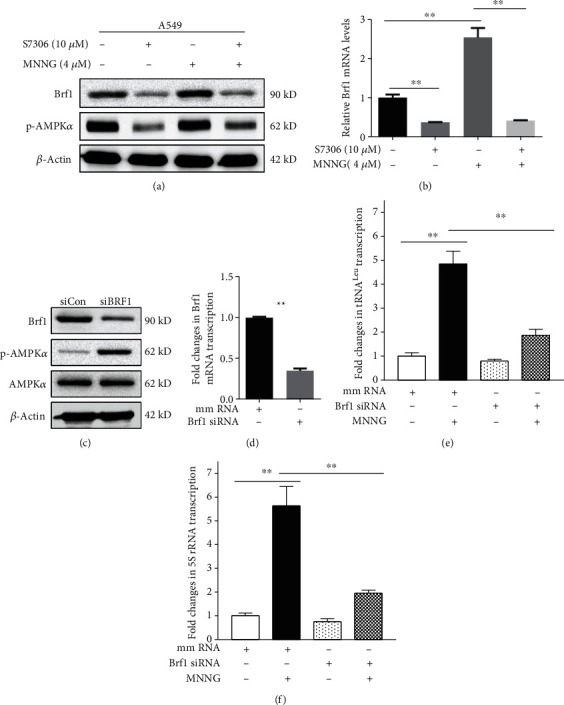
The role of Brf1 alteration in transcription of Pol III genes. (a) Immunoblotting analysis of Brf1 and pAMPK*α* protein levels. A549 cells were treated with AMPK inhibitor, S7306 (12 h with 10 *μ*M), and MNNG (1 h with 4 *μ*M). (b) RT-qPCR analysis of Brf1 mRNA levels in A549 cells treated with S7306 (12 h with 10 *μ*M) and MNNG (1 h with 4 *μ*M). (c) Immunoblotting analysis of Brf1, pAMPK*α*, and AMPK*α* protein levels in MNNG-treated A549 cells after siRNA-mediated knockdown of Brf1, compared to mm siRNA as control (siCon). (d) RT-qPCR analysis of Brf1 mRNA levels in A549 cells which were transfected with mmRNA or Brf1 siRNA to knock down Brf1. (e, f) RT-qPCR analysis. Pol III gene transcription in A549 cells was transfected with Brf1 siRNA or mmRNA for 48 h and then treated with MNNG (4 *μ*M) for 1 h. All error bars represent the SD of at least three independent experiments. *p* values were determined by a two-tailed *t*-test. ^∗^*p* < 0.05, ^∗∗^*p* < 0.01.

**Figure 7:**
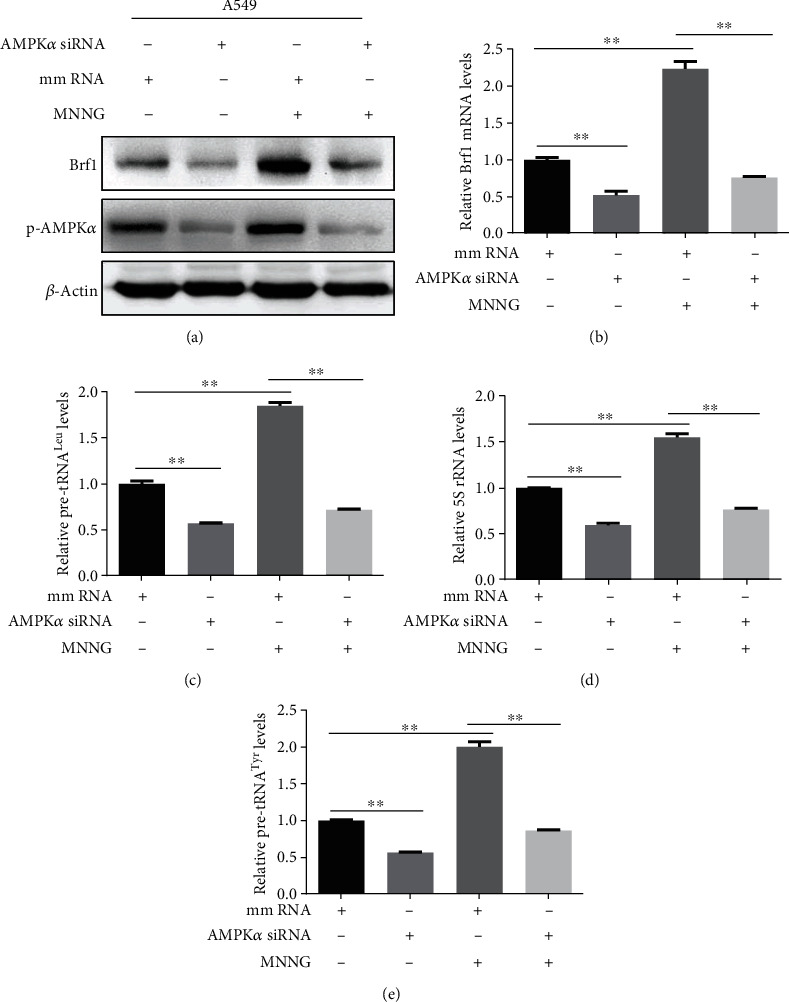
Repressing AMPK*α* expression decreases cellular levels of Brf1 and Pol III genes. (a) Immunoblotting analysis of Brf1 and pAMPK*α* protein levels in A549 cells were treated with MNNG (1 h with 4 *μ*M) after siRNA-mediated knockdown of AMPK*α*. (b) RT-qPCR analysis of Brf1 mRNA levels in A549 cells treated with MNNG (1 h with 4 *μ*M) after siRNA-mediated knockdown of AMPK*α*. (c–e) RT-qPCR analysis. A549 cells were transfected with mmRNA or AMPK*α* siRNA for 48 h and then treated with MNNG (1 h with 4 *μ*M). The cellular levels of pre-tRNA^Leu^ (c), 5S rRNA (d), and pre-tRNA^Tyr^ (e) transcription were determined by RT-qPCR. All error bars represent the SD of at least three replicates from two independent experiments. *p* values were determined by a two-tailed *t*-test. ^∗^*p* < 0.05, ^∗∗^*p* < 0.01.

To further confirm the roles of pAMPK*α* in Brf1 and Pol III gene expression, we pretreated A549 cells with pAMPK*α* specific inhibitor, S7306, and then treated the cells with MNNG as indicated in Figures 6(a) and 6(b). The result displays that S7306 specifically decreases the level of MNNG-induced pAMPK*α* and also reduces the levels of Brf1 protein and mRNA, compared to control cells without the pretreatment by S7306 (Figures 6(a) and 6(b)). This shows that S7306 reduces the activation of AMPK*α* to result in a decrease in MNNG-induced Brf1 expression. Similar results were also observed in H1975 cells (Figures S3A and S3B). Besides, we transfected A549 cells with Brf1 siRNA to repress its expression. The results reveal that Brf1 siRNA can significantly reduce the cellular levels of Brf1, either protein or mRNA (Figures 6(c) and 6(d)), but not the levels of pAMPK*α* and AMPK*α* (Figure 6(c)). Furthermore, our results reveal that repression of Brf1 expression dramatically inhibits the induction of pre-tRNA^Leu^ (Figure 6(e)) and 5S rRNA (Figure 6(f)) caused by MNNG in A549 cells. Our studies have demonstrated that repressing Brf1 expression decreases Pol III gene transcription (Figures 6(e) and 6(f)) [13, 17–19]. Thinking about the effect of nonspecific inhibition of the chemical inhibitor, S7306, we also utilized a specific inhibitor, AMPK*α* siRNA. Compared to control RNA (mismatch RNA, mmRNA), AMPK*α* siRNA markedly reduced the levels of Brf1 protein and mRNAs (Figures 7(a) and 7(b)) and also repressed the transcription of Pol III genes (Figures 7(c)–7(e)). These results (Figures 6 and 7 and Figure S2–3) support the point that pAMPK*α* modulates the expression of Brf1 and Pol III gene transcription.

**Figure 8:**
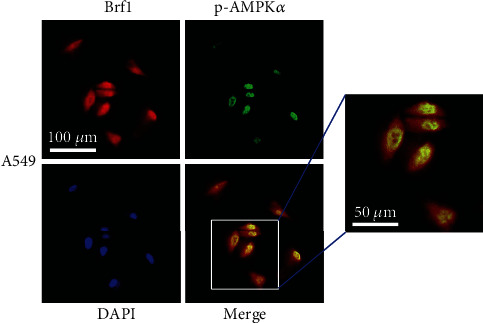
Colocalization of Brf1 and pAMPKα in lung cancer cells. Localizations of Brf1 and pAMPKα: Brf1 (red) and pAMPKα (green) and cell nuclei were stained with DAPI (blue) in A549 cells. The signals of Brf1 (red) and pAMPKα (green) were determined by immunofluorescence staining. The merging picture clearly shows that the colocalization signals of Brf1 and pAMPKα are seen in the nuclei of A549 cells (scale bar = 50 μm).

The above results indicate that AMPK*α* inhibitions (S7306 and AMPK*α* siRNA) reduce the cellular levels of Brf1, leading to decreases in Pol III gene activities (Figures 6 and 7). Therefore, we further determine the colocalization of Brf1 and pAMPK*α* in MNNG-treated lung cancer cells. Immunofluorescent staining indicates that Brf1 reaction with its specific antibody can be observed in the nuclei and plasma of A549 cells (Figure 8(a), red) and H1975 cells (Figure S4, red), while the pAMPK*α* signal is only in the nuclei of the cells (Figure 8(b), green; Figure S4, green). The colocalization signals of Brf1 and pAMPK*α* are seen in the nuclei of the cells (Figure 8, yellow-green and Figure S4). The colocalization of Brf1 and pAMPK*α* implies that pAMPK*α* and Brf1 may synergistically modulate Pol III gene activity [20, 30].

### 3.4. pAMPK*α*-Mediated the Alteration of Brf1 Results in Cellular Phenotypic Changes

**Figure 9:**
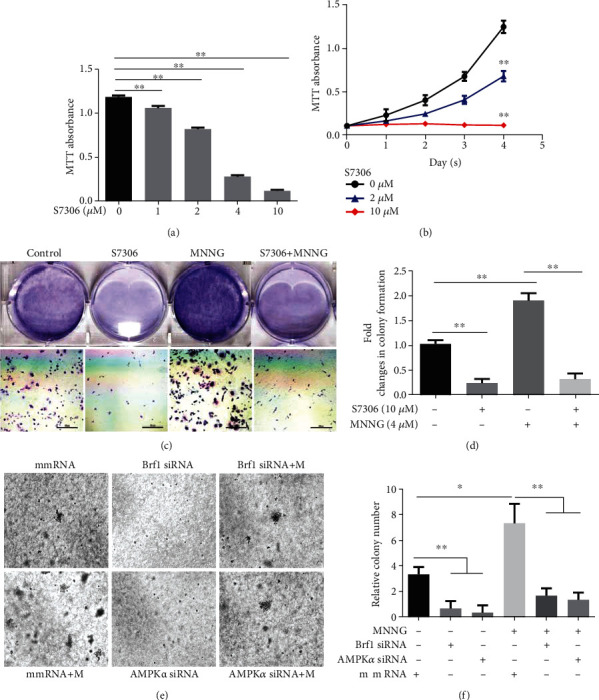
pAMPK*α* mediated the alteration of Brf1 resulting in cellular phenotypic changes. (a, b) MTT assay. A549 cells were pretreated with or without AMPK inhibitor S7306 for 4 days: (a) dose curve (4 days); (b) time course; (c) colony formation assays: A549 cells were cultured in RPMI 1640 alone or with S7306 or MNNG for 1 week or longer (scale bar = 500 *μ*m). The colonies were stained with 0.1% crystal violet solution. (d) Quantification of the colony numbers of A549 cells calculated after being cultured alone or in S7306 or MNNG for 1 week. (e) A549 cells were transfected with mmRNA, Brf1 siRNA, or AMPK*α* siRNA, respectively. After knockdown of Brf1 or AMPK*α* for 48 h, the cells were seeded into soft agar and treated alone or with MNNG (4 *μ*M) for 1-2 weeks to observe colony formation. (f) Quantification of the clonogenicity of A549 cells as described previously (e). All error bars represent the SD of at least three independent experiments. *p* values were determined by a two-tailed *t*-test. ^∗^*p* < 0.05, ^∗∗^*p* < 0.01.

The studies of ours and others have demonstrated that decreasing the expression of Brf1 and Pol III genes represses cell proliferation, cell transformation, and xerograph tumor growth [13, 17–19]. The above results have shown that activated AMPK*α* by the carcinogen MNNG increases the activities of Brf1 and Pol III genes (Figures 5–7, Figure S2). In contrast, inhibiting pAMPK*α* by its specific inhibitor decreases the activities of these genes (Figures 6 and 7 and Figure S3). Therefore, we further determine whether inhibiting AMPK*α* activity causes cellular phenotypic alteration. A549 cells were pretreated with the AMPK*α* inhibitor S7306 and then treated with MNNG to test the changes in cell phenotypes. The results indicate that inhibiting AMPK*α* by S7306 represses the proliferation of A549 cells, compared to control cells (Figures 9(a) and 9(b)). A higher dose of the inhibitor displays more inhibition of cell growth by S7306 (Figure 9(a)).

In addition, we also determined whether inhibiting AMPK*α* reduced the rate of colony formation of A549 cells. The results reveal that MNNG treatment significantly promotes colony formation of the cells, compared to the cells without MNNG treatment (Figure 9(c)), whereas inhibiting AMPK*α* dramatically decreases the rate of colony formation, which displays a significant difference between with and without S7306 treatment (Figure 9(d)). Moreover, we further transfected A549 cells with Brf1 siRNA and AMPK*α* siRNA. The results indicate that repression of either Brf1 or AMPK*α* by their siRNA, the rates of colony formation were significantly attenuated, compared to mm siRNA (Figures 9(e) and 9(f)). These results clearly prove that pAMPK*α* modulates Brf1 expression and Pol III gene transcription, causing cell phenotypic alteration of lung cancer cells.

## 4. Discussion

**Figure 10:**
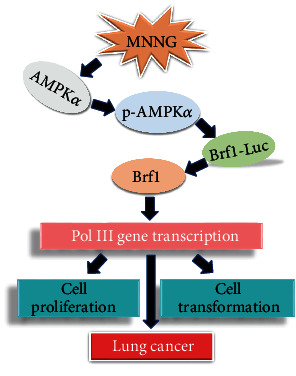
Schematic illustration of pAMPK*α* mediates dysregulation of Brf1 and Pol III gene transcription. In the study, high levels of Brf1 expression and pAMPK*α* are detected in human samples of lung cancer. Mechanism analysis indicates that the carcinogen MNNG induces pAMPK*α* to enhance Brf1 promoter activity, resulting in Brf1 expression and Pol III gene transcription, which increase the rates of cell proliferation and colony formation, eventually resulting in cancer development.

In the present study, we report that Brf1 expression is increased in the cases of human lung cancer. The cases with high Brf1 expression show a short survival period, which means that the prognosis of these cases is worse. Brf1 overexpression in lung cancer cases is accompanied by higher pAMPK*α* levels. Further analysis indicates that pAMPK*α* modulates the activities of Brf1 and Pol III genes. MNNG-increased pAMPK*α* enhances the cellular levels of Brf1 and Pol III gene expression. In contrast, inhibiting AMPK*α* activation reduces Brf1 expression, resulting in decreasing Pol III gene transcription. Brf1 and pAMPK*α* are colocalized in nuclei of lung cancer cells, which suggests that Brf1 and pAMPK*α* may synergistically modulate the activities of Pol III genes [20, 30]. Moreover, inhibiting AMPK*α* activity decreases the rates of proliferation and colony formation of lung cancer cells. These studies, for the first time, demonstrate that overexpression of Brf1 and higher levels of pAMPK*α* are in human lung cancer samples. The activated AMPK*α*, pAMPK*α*, upregulates Brf1 expression and Pol III gene transcription to accelerate cell proliferation and colony formation (Figure 10). These studies indicate that pAMPK*α* and Brf1 play an important role in lung cancer formation.

Brf1 is a key transcription factor. It specifically regulates its target genes, tRNAs and 5S rRNA transcription. Studies have demonstrated that dysregulation of Pol III genes is directly linked to cell transformation and tumorigenesis [16–18]. Upregulation of Pol III genes would serve to enhance the protein biosynthesis to promote cell proliferation and transformation and tumor development and growth, while Brf1 alteration in cells directly affects the products of tRNAs and 5S rRNA genes. Recent studies indicate that Brf1 expression is increased in human cancers of the liver, breast, stomach, and prostate [15, 19–22]. This implies that Brf1 overexpression is required for cancer cell growth in humans. Here, we report that Brf1 expression was enhanced in the cases of lung cancer (Figures 1–3, Figure S1). High Brf1 expression displays a worse prognosis (Figure 2). It suggests that Brf1 is a novel prognostic biomarker for human lung cancer.

LKB1 was originally defined as a tumor suppressor [39]. It is a component of AMPK upstream. Studies have demonstrated that LKB1 mutation in lung cancer is up to 20% or more [26–28]. The mutation of LKB1 often accompanies K-Ras activation in the disease, while activated oncogene, *Ras*, is able to increase the TFIIIB activity to upregulate Pol III gene transcription [39–41]. Brf1 is an important subunit of the TFIIIB complex. This points out that there may be an underlying relationship between Brf1 and lung cancer. Here, we report that Brf1 is overexpressed in cases of lung cancer. It proves the direct relationship between Brf1 and lung cancer. On the other hand, LKB1 activates AMPK activity, while activated AMPK*α* upregulates Brf1 and Pol III gene transcription (Figures 6 and 7) to promote lung cancer development in the status of K-Ras activation [41, 42]. A basic feature of cancer cells is the requirement of high nutrient intakes, macromolecular synthesis, and energy consumption to support tumor cell growth and survival [43]. The biological functions of Brf1 and Pol III genes are responsible for protein synthesis, whereas protein synthesis is essential for tumor cell growth. Eichner and his colleagues reported that AMPK is needed in glucose deprivation to induce Tfe3 activation, while Tfe3 activity increases the growth of rodent lung tumors [42]. Here, our study further demonstrates that the carcinogen MNNG activates AMPK*α* to increase the expression of Brf1 and Pol III genes (Figure 5). In contrast, inhibiting AMPK*α* decreases the expression of these genes (Figures 6 and 7). It shows that there is a new and much more important pathway, namely, pAMPK*α*, which upregulates the activities of Brf1 and Pol III genes to promote human lung cancer development except AMPK-mediated Tfe3.

The studies of signaling events indicate that MAP kinases mediate Pol III gene transcription [39]. Hereafter, JNK1 and JNK2 were identified to differently mediate the activity of Brf1 and Pol III genes: JNK1 positively regulated the activities of Brf1 and Pol III genes to increase cell proliferation [44, 45]. In contrast, JNK2 negatively modulated Brf1 and Pol III genes to repress cell growth [44, 45]. Activated JNK1 upregulates Brf1 expression through the c-Jun and Elk1 pathways to promote liver tumor development [15, 31], whereas activation of JNK1 increases the Brf1 and Pol III gene activities to elevate the rates of breast cell growth and colony formation through ER*α* to facilitate cell transformation [17]. Here, we report that the carcinogen MNNG induces the activation of AMPK*α* to upregulate the expression of Brf1 and Pol III genes, resulting in increasing the rate of cell proliferation and colony formation (Figure 9) [20]. These studies indicate that dysregulation of Brf1 and Pol III genes is going through different signaling pathways in various organs. In other words, the modulations of Brf1 expression are of tissue specificity [14]. Furthermore, we have demonstrated that carcinogens (EGF and DEN) induce histone H3 phosphorylation at serine 10 and 28, while phosphorylated H3 epigenetically upregulates the transcription of Brf1 and Pol III genes [18, 38]. It implies that epigenetic regulation plays a key role in cancer development and tumor growth. These studies have been going in our laboratory.

## 5. Conclusion

In summary, our studies demonstrate that Brf1 is overexpressed in human lung cancer. High Brf1 expression displays short survival times. The overexpression of Brf1 is accompanied by a high level of pAMPK*α* in the cases of lung cancer. Mechanism study reveals that activated AMPK*α*, pAMPK*α*, upregulates the activities of Brf1 and Pol III genes, while repressing AMPK*α* decreases Brf1 expression and Pol III gene transcription, resulting in the reduction of cell proliferation and colony formation (Figure 10) [29]. These studies demonstrate that pAMPK*α*-mediated Brf1 expression and Pol III gene transcription is a novel and direct pathway, which is tightly linked to protein synthesis, supporting cell growth and cell survival of lung cancer. Therefore, developing a specific inhibitor to repress the growth of cancer cells is a new strategy for the therapy of human lung cancer.

## Figures and Tables

**Table 1:** Correlation between Brf1 expression and clinicopathological features in 226 primary lung cancers.

Parameters	Low Brf1 expression	High Brf1 expression	Chi-squared test *p* value	Fisher's exact test *p* value
	*N* = 89 (39.4%)	*N* = 137 (60.6%)		
Age				
<48	7 (7.9%)	6 (4.4%)		
≥48	82 (92.1%)	131 (95.6%)	0.272	0.381
Gender				
Male	53 (59.6%)	114 (83.2%)		
Female	36 (40.4%)	23 (16.8%)	0.000	0.000
Differentiation status				
Well/moderate	53 (59.6%)	77 (56.2%)		
Poor and others	36 (40.4%)	60 (43.8%)	0.619	0.680
Lymph node invasion (N stage)				
Absent	72 (80.9%)	91 (66.4%)		
Present	17 (19.1%)	46 (33.6%)	0.018	0.022
Clinical stage				
I, II	27 (30.3%)	75 (54.7%)		
III, IV	62 (69.7%)	62 (45.3%)	0.000	0.000

^∗^ *p* values determined by using SPSS 20.0. All statistical tests were two-sided.

**Table 2:** Effect of factors on overall survival in lung cancer patients in the univariate and multivariate Cox regression model.

Factors	Univariate^∗^		Multivariate^∗^^,†,‡^	
	HR (95% CI)	*p*	HR (95% CI)^∗^	*p*
Age (<48/≥48)	2.69 (0.85-8.49)	0.091	—	—
Gender (male/female)	1.34 (0.85-2.12)	0.206	—	—
Differentiation (poor/well, moderate)	1.12 (0.76-1.65)	0.563	—	—
Lymph node invasion (present/absent)	0.49 (0.33-0.71)	0.000	—	—
Clinical stage (III-IV/I-II)	2.85 (1.92-4.22)	0.000	2.71 (1.82-4.02)	0.000
Brf1 (high/low)	0.55 (0.36-0.84)	0.006	0.60 (0.39-0.93)	0.021

^∗^Hazard ratios and *p* values were obtained from Cox proportional hazards regression. All statistical tests were two-sided. ^†^For the multivariate model, HR and *p* values were shown for the final set of stepwise selected variables only. ^‡^The parameters with *p* value less than 0.05 in the univariate were included in the multivariate Cox analysis using SPSS 20.0.

## Data Availability

The datasets of the study are available on request to the corresponding authors.

## Abbreviations

AMPK: 5′ AMP-activated protein kinase
pAMPK*α*: Phosphorylated AMPK*α*
Brf1: TF IIB-related factor 1
Pol III genes: RNA polymerase III-dependent genes
TFIIIB: Transcription factor III B complex
SCLC: Small-cell lung cancer
NSCLC: Non-small-cell lung cancer
LKB1: Tumor suppressor liver kinase B1
IHC: Immunohistochemistry
ANT: Adjacent noncancerous tissue
SI: Staining index
mm siRNA: Mismatch siRNA
RT-qPCR: Real-time quantitative PCR
Brf1-Luc: Brf1 promoter-luciferase reporter
ER*α*: Estrogen receptor *α*
MNNG: N-Methyl-N′-nitro-N-nitrosoguanidine
ADH: Alcohol dehydrogenase
CYP2E1: Cytochrome P450 2E1
ROS: Reactive oxygen species.

## Supplementary Materials

Supplementary Materials Supplementary figures (Figure S1: immunohistochemical staining in the samples of lung cancer patients. Figure S2: carcinogen MNNG increases Brf1 expression and Pol III gene transcription. Figure S3: the roles of AMPK*α* in Brf1 expression in lung cancer cells. Figure S4: colocalization of Brf1 and pAMPK*α* in lung cancer cells.) are available at *Oxidative Medicine and Cellular Longevity* online.

## References

[1] Wu Y. L., Cheng Y., Zhou X. (2017). Dacomitinib versus gefitinib as first-line treatment for patients with _EGFR_ -mutation-positive non-small-cell lung cancer (ARCHER 1050): a randomised, open-label, phase 3 trial. *The Lancet Oncology*.

[2] Chen W., Zheng R., Baade P. D. (2016). Cancer statistics in China, 2015. *CA: a Cancer Journal for Clinicians*.

[3] Reguart N., Marin E., Remon J., Reyes R., Teixido C. (2020). In search of the long-desired 'Copernican therapeutic revolution' in small-cell lung cancer. *Drugs*.

[4] Davidson M. R., Gazdar A. F., Clarke B. E. (2013). The pivotal role of pathology in the management of lung cancer. *Journal of Thoracic Disease*.

[5] Dayen C., Debieuvre D., Molinier O. (2017). New insights into stage and prognosis in small cell lung cancer: an analysis of 968 cases. *Journal of Thoracic Disease*.

[6] Aviel-Ronen S., Blackhall F. H., Shepherd F. A., Tsao M. S. (2006). K- _ras_ mutations in non-small-cell lung carcinoma: a review. *Clinical Lung Cancer*.

[7] Cooper W. A., Lam D. C., O'Toole S. A., Minna J. D. (2013). Molecular biology of lung cancer. *Journal of Thoracic Disease*.

[8] Herbst R. S., Heymach J. V., Lippman S. M. (2008). Lung cancer. *The New England Journal of Medicine*.

[9] Takahashi N., Chen H. Y., Harris I. S. (2018). Cancer cells co-opt the neuronal redox-sensing channel TRPA1 to promote oxidative-stress tolerance. *Cancer Cell*.

[10] Vlahopoulos S., Adamaki M., Khoury N., Zoumpourlis V., Boldogh I. (2019). Roles of DNA repair enzyme OGG1 in innate immunity and its significance for lung cancer. *Pharmacology & Therapeutics*.

[11] Jakopovic M., Thomas A., Balasubramaniam S., Schrump D., Giaccone G., Bates S. E. (2013). Targeting the epigenome in lung cancer: expanding approaches to epigenetic therapy. *Frontiers in Oncology*.

[12] White R. J. (2004). RNA polymerase III transcription and cancer. *Oncogene*.

[13] Johnson D. L., Johnson S. A. (2008). Cell biology. RNA metabolism and oncogenesis. *Science*.

[14] Huang C., Zhang Y., Zhong S. (2019). Alcohol intake and abnormal expression of Brf1 in breast cancer. *Oxidative Medicine and Cellular Longevity*.

[15] Shi G., Zhong S. (2017). Alcohol-associated cancer and deregulation of Pol III genes. *Gene*.

[16] Johnson S. A., Dubeau L., Johnson D. L. (2008). Enhanced RNA Polymerase III-dependent Transcription Is Required for Oncogenic Transformation∗. *The Journal of Biological Chemistry*.

[17] Zhang Q., Jin J., Zhong Q., Yu X., Levy D., Zhong S. (2013). ER*α* mediates alcohol-induced deregulation of Pol III genes in breast cancer cells. *Carcinogenesis*.

[18] Zhong Q., Shi G., Zhang Q., Zhang Y., Levy D., Zhong S. (2013). Role of phosphorylated histone H3 serine 10 in DEN-induced deregulation of Pol III genes and cell proliferation and transformation. *Carcinogenesis*.

[19] Zhong Q., Xi S., Liang J. (2016). The significance of Brf1 overexpression in human hepatocellular carcinoma. *Oncotarget*.

[20] Fang Z., Yi Y., Shi G. (2017). Role of Brf1 interaction withER*α*, and significance of its overexpression, in human breast cancer. *Molecular Oncology*.

[21] Zhang Y., Wu H., Yang F. (2018). Prognostic value of the expression of DNA repair-related biomarkers mediated by alcohol in gastric cancer patients. *The American Journal of Pathology*.

[22] Loveridge C. J., Slater S., Campbell K. J. (2020). BRF1 accelerates prostate tumourigenesis and perturbs immune infiltration. *Oncogene*.

[23] Stapleton D., Mitchelhill K. I., Michell B. J. (1996). Mammalian AMP-activated protein kinase subfamily (∗). *The Journal of Biological Chemistry*.

[24] Jeon S. M. (2016). Regulation and function of AMPK in physiology and diseases. *Experimental & Molecular Medicine*.

[25] Hardie D. G., Ross F. A., Hawley S. A. (2012). AMPK: a nutrient and energy sensor that maintains energy homeostasis. *Nature Reviews Molecular Cell Biology*.

[26] Chen Z., Cheng K., Walton Z. (2012). A murine lung cancer co-clinical trial identifies genetic modifiers of therapeutic response. *Nature*.

[27] Han X., Li F., Fang Z. (2014). Transdifferentiation of lung adenocarcinoma in mice with _Lkb1_ deficiency to squamous cell carcinoma. *Nature Communications*.

[28] Ji H., Ramsey M. R., Hayes D. N. (2007). LKB1 modulates lung cancer differentiation and metastasis. *Nature*.

[29] Hong Z., Lin M., Zhang Y., He Z., Zheng L., Zhong S. (2020). Role of betaine in inhibiting the induction of RNA Pol III gene transcription and cell growth caused by alcohol. *Chemico-Biological Interactions*.

[30] Hong Z., Fang Z., Lei J. (2020). The significance of Runx2 mediating alcohol-induced Brf1 expression and RNA Pol III gene transcription. *Chemico-Biological Interactions*.

[31] Zhong S., Machida K., Tsukamoto H., Johnson D. L. (2011). Alcohol induces RNA polymerase III-dependent transcription through c-Jun by co-regulating TATA-binding protein (TBP) and Brf1 expression∗. *The Journal of Biological Chemistry*.

[32] Lin M., Huang C., Ren W., Chen J., Xia N., Zhong S. (2020). Mitogen- and stress-activated protein kinase 1 mediates Alcohol-upregulated transcription of Brf1 and tRNA genes to cause phenotypic alteration. *Oxidative Medicine and Cellular Longevity*.

[33] Purohit V., Khalsa J., Serrano J. (2005). Mechanisms of alcohol-associated cancers: introduction and summary of the symposium. *Alcohol*.

[34] Shan C., Lu Z., Li Z. (2019). 4-Hydroxyphenylpyruvate dioxygenase promotes lung cancer growth via pentose phosphate pathway (PPP) flux mediated by LKB1-AMPK/HDAC10/G6PD axis. *Cell Death & Disease*.

[35] Li S., Zhuang Z., Wu T. (2018). Nicotinamide nucleotide transhydrogenase-mediated redox homeostasis promotes tumor growth and metastasis in gastric cancer. *Redox Biology*.

[36] Li W., Yu C. P., Xia J. T. (2009). Sphingosine kinase 1 is associated with gastric cancer progression and poor survival of patients. *Clinical Cancer Research*.

[37] Li S., Wu T., Lu Y. X. (2020). Obesity promotes gastric cancer metastasis via diacylglycerol acyltransferase 2-dependent lipid droplets accumulation and redox homeostasis. *Redox Biology*.

[38] Zhang Q., Zhong Q., Evans A. G., Levy D., Zhong S. (2011). Phosphorylation of histone H3 serine 28 modulates RNA polymerase III- dependent transcription. *Oncogene*.

[39] Hemminki A., Markie D., Tomlinson I. (1998). A serine/threonine kinase gene defective in Peutz-Jeghers syndrome. *Nature*.

[40] Zhong S., Zhang C., Johnson D. L. (2004). Epidermal growth factor enhances cellular TATA binding protein levels and induces RNA polymerase I- and III-dependent gene activity. *Molecular and Cellular Biology*.

[41] Johnson S. A., Dubeau L., Kawalek M. (2003). Increased expression of TATA-binding protein, the central transcription factor, can contribute to oncogenesis. *Molecular and Cellular Biology*.

[42] Eichner L. J., Brun S. N., Herzig S. (2019). Genetic analysis reveals AMPK is required to support tumor growth in murine Kras-dependent lung cancer models. *Cell Metabolism*.

[43] Cairns R. A., Harris I. S., Mak T. W. (2011). Regulation of cancer cell metabolism. *Nature Reviews Cancer*.

[44] Zhong and S., Johnson D. L. (2009). The JNKs differentially regulate RNA polymerase III transcription by coordinately modulating the expression of all TFIIIB subunits. *Proceedings of the National Academy of Sciences of the United States of America*.

[45] Zhong S., Fromm J., Johnson D. L. (2007). TBP is differentially regulated by c-Jun N-terminal kinase 1 (JNK1) and JNK2 through Elk-1, controlling c-Jun expression and cell proliferation. *Molecular and Cellular Biology*.

