# Supplementary material for: Exploring the Role and Mechanism of pAMPKα-Mediated Dysregulation of Brf1 and RNA Pol III Genes
Source: Oxid Med Cell Longev. 2021 Apr 20;2021:5554932. doi: 10.1155/2021/5554932 (PMC8081602; doi:10.1155/2021/5554932)
Supplement: Supplementary Materials — Supplementary figures (Figure S1: immunohistochemical staining in the samples of lung cancer patients. Figure S2: carcinogen MNNG increases Brf1 expression and Pol III gene transcription. Figure S3: the roles of AMPKα in Brf1 expression in lung cancer cells. Figure S4: colocalization of Brf1 and pAMPKα in lung cancer cells.) are available at Oxidative Medicine and Cellular Longevity online. [file 5554932.f1.pdf]

Supplements

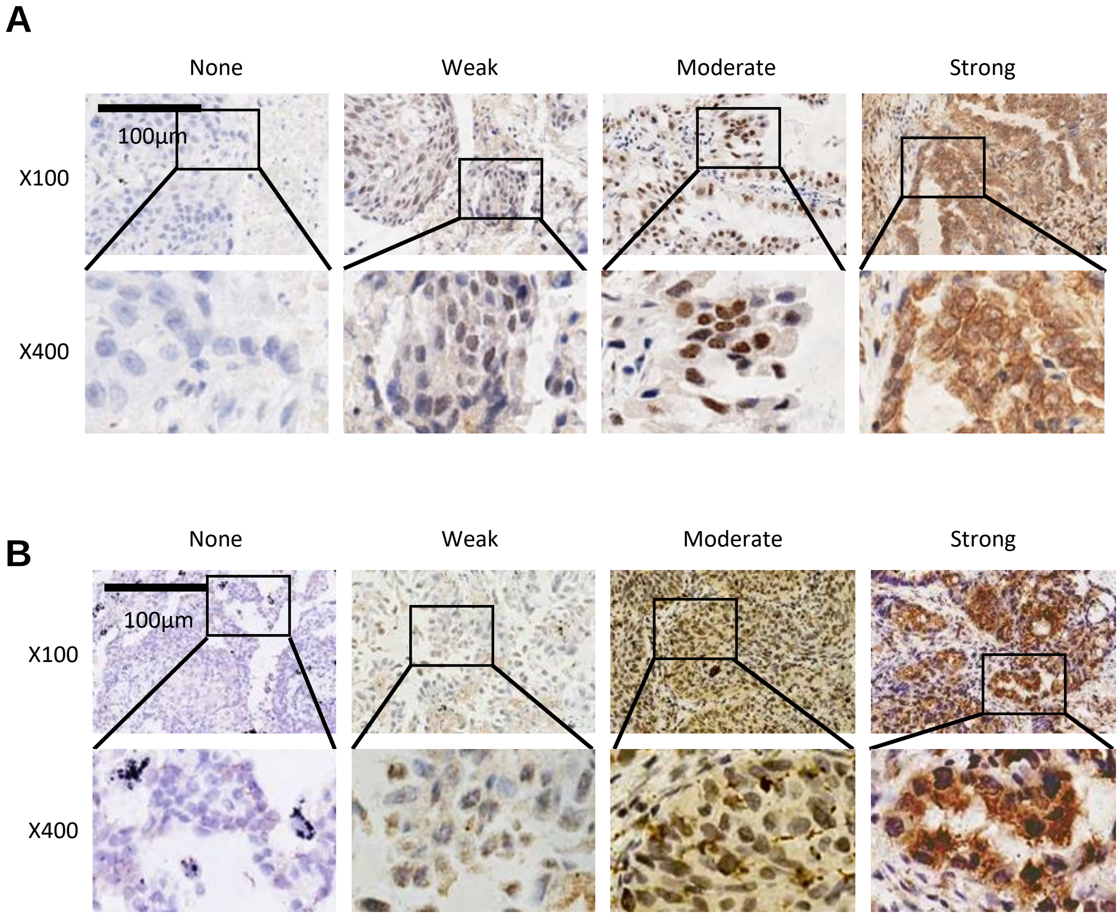

Figure S1. Immunohistochemical staining in the samples of lung cancer patients. (A and B) *Brf1 signals in different tissues of lung cancer.* In terms of the staining intensity of Brf1, IHC staining signals of Brf1 expression are divided into four groups from left side to right side: None is no any signal in non-tumor tissues; Weak is lightly staining signals of Brf1 in tumor tissue; Moderate is obvious staining signals; Strong is dramatic intensity of Brf1 expression in tumor tissues. Magnification, **A and B upper panel**: 100X, (scale bar=100µm); Magnification, **A and B bottom panel:** 400 X.

Fig. S1

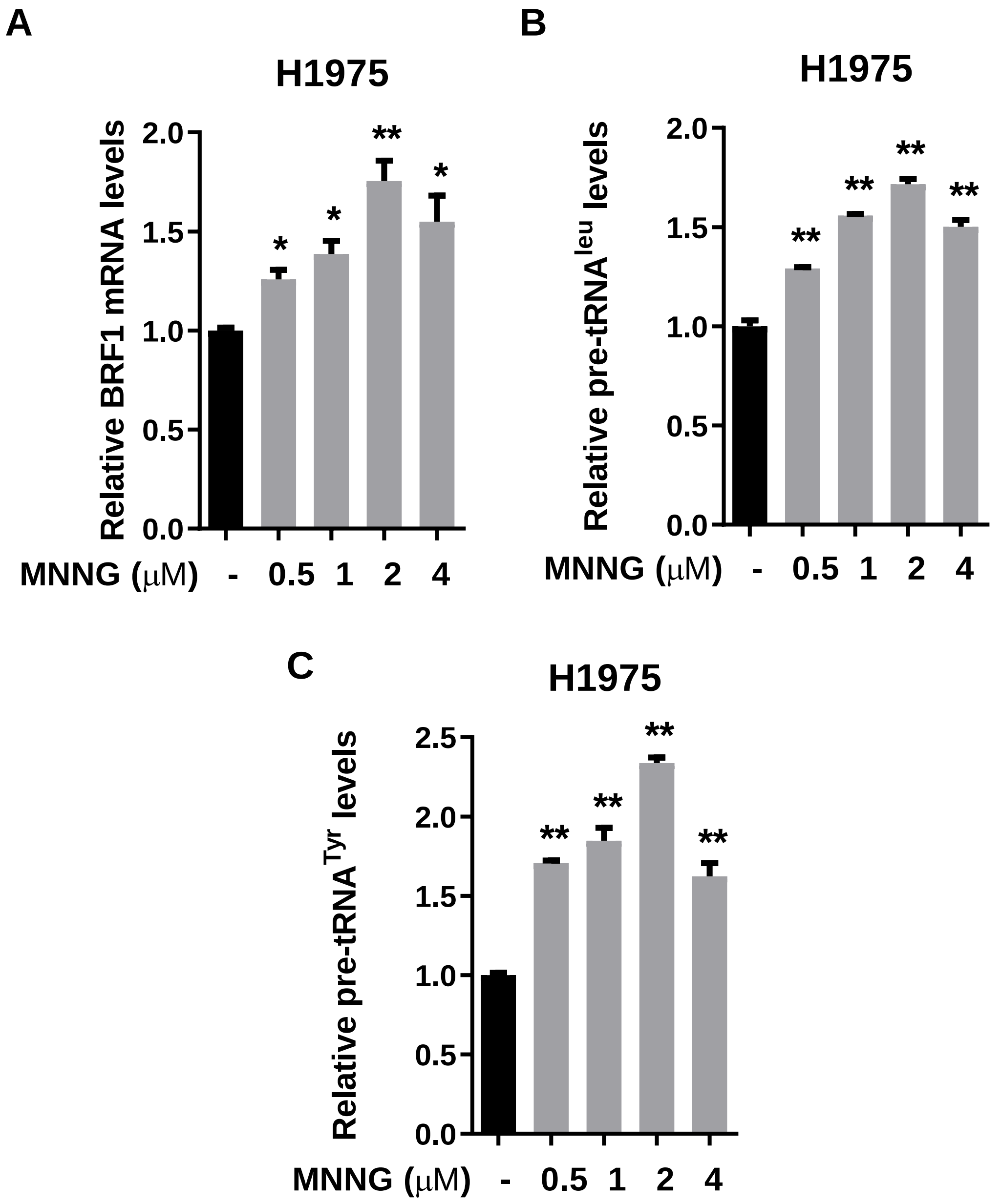

Figure S2. Carcinogen, MNNG increases Brf1 expression and Pol III gene transcription. H1975 cells were treated with different doses of carcinogen, MNNG as described. The resultant total RNA were extracted from the cells to determine the levels of Brf1 mRNA and Pol III gene transcription by RT-qPCR. **(A)** Brf1 mRNA; **(B)** $tRNA^{Leu}$; **(C)** $tRNA^{Tyr.}$ The results indicate that MNNG enhanced Brf1 expression and Pol III gene transcription. All error bars represent the SD at least three independent experiments. *P* values were determined by two-tailed t test. **P*< 0.05, ***P*< 0.01.

Fig. S2

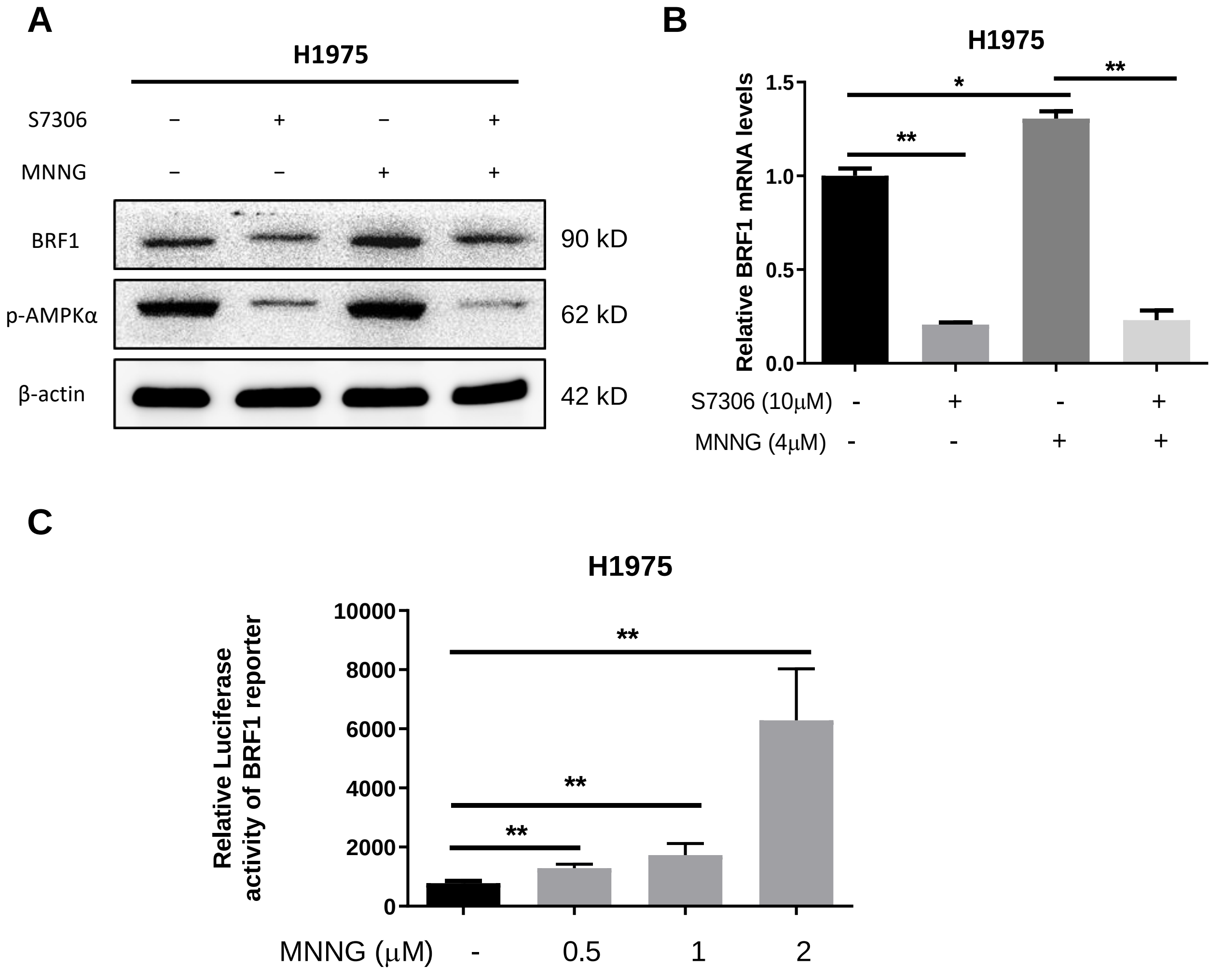

Figure S3 The roles of AMPKα in Brf1 expression in lung cancer cells. H1975 cells were pretreated with AMPK specific inhibitor, S7306 and then treated with MNNG to extract cell lysates and total RNA as described above. **(A)** Immunoblotting analysis of Brf1 and pAMPKα protein levels. The resultant cell lysates were used to determine the levels of Brf1 protein and pAMPKα. **(B)** RT-qPCR analysis of Brf1 mRNA levels in H1975 cells. **(C)** Brf1 promoter luciferase activity. The H1975 cells were transfected with 0.5μg Brf1-Luc plasmids. Luciferase assay indicates that carcinogen, MNNG increases the activity of Brf1 promoter. All error bars represent the SD of at least three independent experiments. *P* values were determined by two-tailed t test. *$P < 0.05$, **$P < 0.01$.

Fig. S3

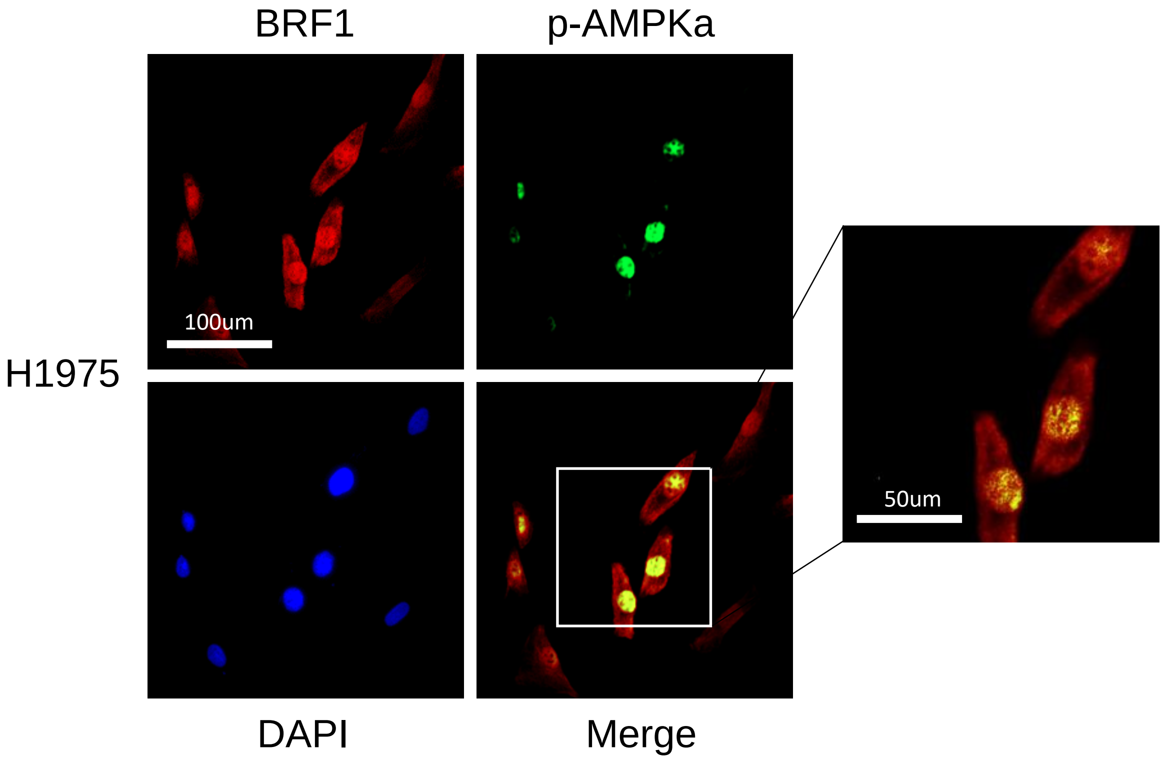

Figure S4. Colocalization of Brf1 and pAMPKα in lung cancer cells. Colocalization of Brf1 and pAMPKα: Brf1 (**Red**) and pAMPKα (**Green**) and cell nuclei were stained with DAPI (**Blue**) in H1975 cells were determined by immunofluorescence staining. Merging picture clearly shows that the localization signals of Brf1 and pAMPKα are seen in nuclei of A549 cells. (scale bar=50μm).

Fig. S4
